# Promising MPPT Methods Combining Metaheuristic, Fuzzy-Logic and ANN Techniques for Grid-Connected Photovoltaic †

**DOI:** 10.3390/s21041244

**Published:** 2021-02-10

**Authors:** Mahmoud N. Ali, Karar Mahmoud, Matti Lehtonen, Mohamed M. F. Darwish

**Affiliations:** 1 Department of Electrical Engineering, Faculty of Engineering at Shoubra, Benha University, Cairo 11629, Egypt; mahmoud.nour@feng.bu.edu.eg; 2 Department of Electrical Engineering and Automation, Aalto University, 02150 Espoo, Finland; karar.mostafa@aalto.fi (K.M.); matti.lehtonen@aalto.fi (M.L.); 3 Department of Electrical Engineering, Aswan University, Aswan 81542, Egypt

**Keywords:** PV system, maximum power point tracking, artificial intelligence, fuzzy logic control, artificial neural network, genetic algorithm, particle swarm optimization

## Abstract

This paper addresses the improvement of tracking of the maximum power point upon the variations of the environmental conditions and hence improving photovoltaic efficiency. Rather than the traditional methods of maximum power point tracking, artificial intelligence is utilized to design a high-performance maximum power point tracking control system. In this paper, two artificial intelligence-based maximum power point tracking systems are proposed for grid-connected photovoltaic units. The first design is based on an optimized fuzzy logic control using genetic algorithm and particle swarm optimization for the maximum power point tracking system. In turn, the second design depends on the genetic algorithm-based artificial neural network. Each of the two artificial intelligence-based systems has its privileged response according to the solar radiation and temperature levels. Then, a novel combination of the two designs is introduced to maximize the efficiency of the maximum power point tracking system. The novelty of this paper is to employ the metaheuristic optimization technique with the well-known artificial intelligence techniques to provide a better tracking system to be used to harvest the maximum possible power from photovoltaic (PV) arrays. To affirm the efficiency of the proposed tracking systems, their simulation results are compared with some conventional tracking methods from the literature under different conditions. The findings emphasize their superiority in terms of tracking speed and output DC power, which also improve photovoltaic system efficiency.

## 1. Introduction

Recently, photovoltaic (PV) systems have been used intensively in distribution networks worldwide to generate electric power from sunlight beside the load centers. The total worldwide capacity of PV has experienced approximately exponential progress in the earlier decades, cumulative from 39 GWp in 2010 to 480 GWp in 2018 while the typical PV installation costs reducing from 4621 USD/kWp to 1210 USD/kWp for the same duration [1]. European Union (EU) follows an ambitious strategy to be the world leader in the sector of renewable energy by 2030 [2]. For example, the share of renewables in Finland is 47% of all generation in 2018, including wind, PV, and Hydropower stations [3]. In general, these systems exist in remote regions, i.e., standalone, or in grid-connected units. In this regard, the PV efficiency is mostly dependent on their operation at maximum power point (MPP) during different grid conditions. The MPP tracking (MPPT) is considered a control unit to preserve the output DC generated power at the maximum rate at numerous environmental and load circumstances [4,5,6,7]. A major benefit of efficiency improvement of PV is to minimize the overall operating cost of using these distributed generations (DG), which is a challenge towards the effective utilization of renewable DG units [8,9,10,11,12,13,14].

Recently, diverse MPPT schemes have been extensively proposed for solving the MPP uniqueness in numerous applications. Typically, the control systems of PV depend mainly on the DC-DC boost converter, which adjusts the duty cycle during environmental conditions fluctuations (i.e., cell temperature and irradiance). Common methods for MPPT of PV involve (1) incremental conductance (INC) method, (2) perturb-and-observe (P&O) method, and (3) the Hill Climbing method, have been implemented in [15,16]. More recently, the employment of advanced artificial intelligence (AI) is expanded in various subjects which can also be employed in the MPPT of grid-connected PV units [17]. Advanced MPPT approaches have been also adopted such as the genetic algorithm (GA) [18] and the fuzzy logic control (FLC) [19,20]. Driven by the advanced innovations in metaheuristic optimization, different variants have been employed in the MPPT problem, like ant colony optimization algorithm [21], particle swarm optimization [22], and differential evaluation [23]. Further metaheuristic based approaches for the MPPT control of PV involve cuckoo Search Algorithm [24], Jaya optimizer [25], and spline model guided MPPT method [26]. Other methods exploit the common methods and AI techniques as hybrid MPPT control systems [27]. The authors of [28] evaluates classical techniques based on PV efficiency is investigated. Comparative MPPT studies by the common and AI techniques have been presented in [29,30], which have highlighted the features of employing the advanced algorithms. Further, artificial neural networks (ANNs) have been extensively utilized in different areas as rapid, precise, and robust tools due to their effective learning schemes [31,32,33]. Specifically, ANNs can simplify complex mathematical models by the dense connections among the neurons. For the purpose of MPPT, ANNs are used with dissimilar architectures and input signals considering different grid and environmental situations [34,35,36,37]. Regarding ANN based MPPT units, the commonly used input signals are the irradiance and the cell temperature. In [38], the GA optimization has been integrated with ANN to enhance the operation of a standalone PV system by using the two common input signals. The authors of [39] have proposed the use of GA and ANN to refining the search procedure for MPPT. Another application of GA is to optimize the training dataset of ANN for MPPT of PV systems [18]. In a previous work reported in [40,41], an ANN-based MPPT method supported with GA has been proposed for PV systems.

As illustrated in the literature, several methods have been used for MPPT of PV systems. To cover the limitations of the existing methods, in this paper, two AI-based MPPT systems are introduced for grid-connected PV. The first AI system is based on an enhanced fuzzy logic control (FLC) by means of GA and particle swarm optimization (PSO). Further, the second one depends on the GA-based ANN (called GA-ANN). Each of the two AI-based MPPT systems has its superior response based on solar radiation and temperature. Most importantly, a new combination of these two AI designs is proposed to maximize the efficiency of the MPPT system of PV. According to the authors’ knowledge, this is the first paper to combine GA, PSO, ANN, and FLC to efficiently address the MPPT for PV systems. Comprehensive simulation results are compared with some common MPPT methods from the literature under different conditions to affirm the efficiency of the three proposed MPPT systems. It is proven that the proposed systems have superior performances in terms of tracking speed and output DC power, thereby improving the PV system efficiency.

The novelty of this work is to utilize the metaheuristic optimization technique with the well-known artificial intelligence methods to achieve a better tracking system that harvests the maximum possible power from PV systems. GA and PSO are exploited to adapt the FLC system for MPPT. In turn, the GA is exploited to assist the choice of the suitable ANN architecture for MPPT. As it was remarked, each of the two MPPT systems acts well at different irradiation and temperature range. So, a proposed combination of the two AI-based systems is presented to exploit the superiority of each of them for MPPT. Specifically, we introduce a novel AI-based MPPT method that combines Fuzzy logic controller and ANN to exploits the best of them. The detailed contributions are as follows:

- Using of Fuzzy logic controller as MPPT system optimized by GA and PSO solvers;
- Using GA for design the architecture of ANN-based MPPT;
- Comparison between these two AI-based methods;
- Proposition a combination of the two methods because each of them is better for a certain range of irradiance and temperature;
- The results are elaborated and comparisons with incremental conductance and perturb and observe methods are presented;
- The comparisons are presented for both linear and step variations of irradiance and temperature.

The remainder of the paper is structured as follows. Section 2.1 assesses the PV array modeling and Section 2 presents the methods of maximum power point tracking. In Section 3, the application of the artificial intelligence methods for MPPT is illustrated where the results. In Section 3.5, a discussion of the main findings in the paper are discussed. Finally, Section 4 summarizes the paper, emphasizing its main conclusions.

## 2. Methods of Maximum Power Point Tracking

### 2.1. PV Array Modeling

**Figure 1:**
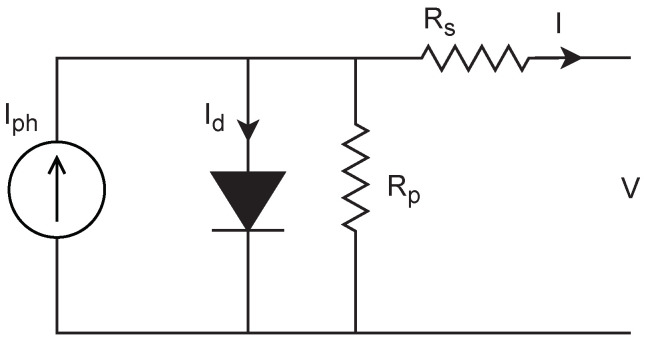
The model of a photovoltaic (PV) cell: one diode model.

The connection of PV cells to constitute PV panels and arrays relies on the needed power and voltage. The PV cell modeling, which has different modeling approaches, represents the stone for the PV array modeling. The PV cell model that satisfies the modeling requirements and simplicity is the one diode model presented in Figure 1. The current (I)-voltage (V) relations of a PV cell can be presented as follows [42,43]:

(1) $$\begin{array}{c} I=I_{ph}-I_{s}\left(exp\left(\frac{q(V+IR_{s})}{akT_{c}}-1\right)\right)-\frac{V+IR_{s}}{R_{p}} \end{array}$$

where, $I_{ph}$ is the photo current and $I_{s}$ is the saturation current, which are given as [42,43]:

(2) $$I_{ph}=\frac{G}{G_{n}}\left(I_{sc_{n}}+K_{I}\left(T_{c}-T_{c_{n}}\right)\right)$$

(3) $$I_{s}=I_{s_{n}}{\left(\frac{T_{c_{n}}}{T_{c}}\right)}^{3}exp\left(\frac{qE_{g}}{ak}\left(1/T_{c_{n}}-1/T_{c}\right)\right)$$

(4) $$I_{s_{n}}=\frac{I_{sc_{n}}}{exp(\frac{qV_{oc_{n}}}{akT_{c_{n}}})-1}$$

where, *G* is the solar irradiance in W/m${}^{2}$, $T_{c}$ is the cell temperature in Kelvin, $V_{oc}$ is the open circuit voltage in *V*, $I_{sc}$ is the short circuit current in *A*, $R_{s}$ is the series resistance, $R_{p}$ is the parallel resistance, $E_{g}$ is the band gap in *J*, *k* is the Boltzmann constant, *a* is the ideality factor and *q* is the electron charge [42,43]. The standard test conditions case, where $G_{n}=1000$ W/m${}^{2}$, $T_{c_{n}}=25^{\;\circ }$C, is denoted by the subscript *n*.

**Figure 2:**
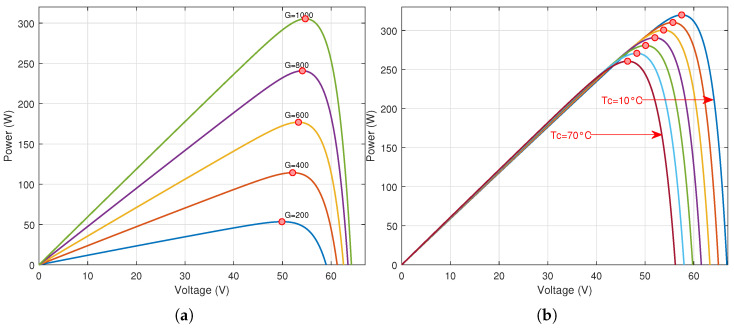
The change of the maximum power point of the PV panel (SUNPOWER 305) for the variation of: (**a**) solar irradiance at $T_{c}=25^{\;\circ }$C; (**b**) cell temperature at G=1000 W/m${}^{2}$.

To illustrate the need of the control systems to track the maximum power point when the environmental circumstances change, the relations between the PV voltage and the output DC power of a PV panel is presented. These relations affirm that when the environmental conditions, e.g., *G* and $T_{c}$, change, the point of maximum power, which is the optimal point of operation, change. Therefore, an accurate MPPT system is needed to preserve the optimal operation of PV systems. Figure 2 presents the voltage power relation for a PV panel, with varying *G* and $T_{c}$. The simulated PV panel is the SUNPOWER 305.

### 2.2. Conventional Methods

The incremental conductance (INC) and the perturb-and-observe (P&O) methods are the most widely used conventional MPPT methods. The INC method is based on the slope of the voltage-power relation, which represents the optimal operation point (maximum output power) when reaching zero. If this slope tends to be positive, the PV voltage requires to be increased and if the slope is negative, the PV voltage requires to be decreased as the following equations summarize [29,30]:

(5) P=VI

(6) $$\frac{dP}{dV}=I+\frac{VdI}{dV}$$

where, *V* is the PV voltage, *I* is the PV current and *P* is the output DC power of a PV panel. At maximum power, dP/dV=0, which leads to:

(7) $$\frac{I}{V}=-\frac{dI}{dV}$$

When $\frac{dP}{dV}>0$, i.e., $I>-\frac{VdI}{dV}$, the voltage requires to be increased, and when $\frac{dP}{dV}<0$, i.e., $I<-\frac{VdI}{dV}$, the voltage needs to be decreased.

The conventional P&O method depends on the perturbation of the PV voltage/duty cycle, with a fixed feasible step size and observe the corresponding output DC power. If an increase of power is observed, additional perturbation in the same direction is effectuated, otherwise, its direction is reversed. More details about these two conventional methods can be found in [15,42,44]. These two method are introduced as reference methods for comparison with the improved artificial intelligence based MPPT methods.

### 2.3. Artificial Intelligence Methods for MPPT

#### 2.3.1. GA/PSO Fuzzy Logic MPPT

**Figure 3:**
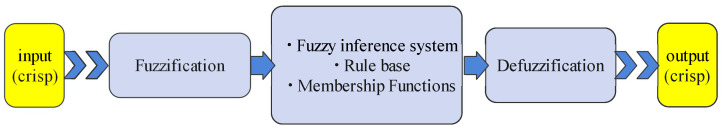
The basic stages in a fuzzy logic controller.

The fuzzy logic controller (FLC) is used for different control systems having uncertainties due to its independence of mathematical models. The basic stages of a fuzzy controller is shown in Figure 3. The input and output of the fuzzy system are crisp. The crisp input is converted to a fuzzy input through the fuzzification process based on the type and degree of membership function used. The rule base is a set of *if-then* rules, which may be extracted from human experience or from automatic rule generation. In the fuzzy inference stage and depending on the fuzzy rules, an implication method and an aggregation of all fuzzy outputs are applied to get the overall output fuzzy variable. To get the crisp output used in the control process, the defuzzification process is applied [45].

**Figure 4:**
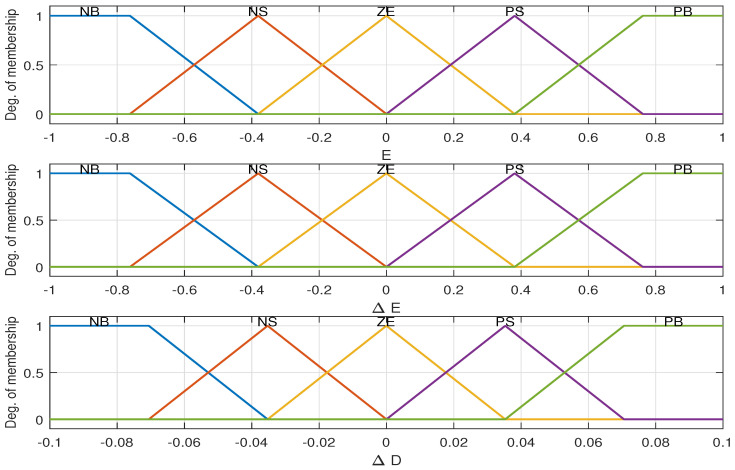
The membership functions of the fuzzy logic control (FLC) inputs (*E* and ΔE) and output (ΔD).

The FLC was effectively used with different configuration for MPPT system [46,47]. For this paper, the fuzzy inputs chosen are the error signal *E*, where ($E=\frac{P_{k}-P_{k-1}}{V_{k}-V_{k-1}}$), and the change of this error ($\Delta E=E_{k}-E_{k-1}$). The designed objective from FLC based MPPT is a change in the PV voltage (increment or decrement), which is achieved through the change of the duty cycle (ΔD), which is the output FLC. The initial membership functions used for the inputs and the output of FLC based MPPT is shown in Figure 4.

**Figure 5:**
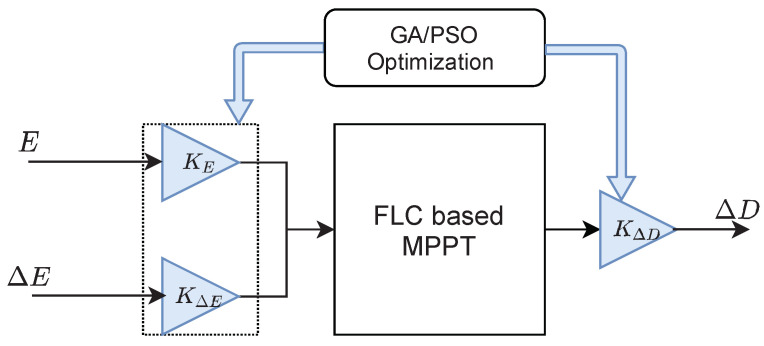
Optimization of the FLC gains.

It was always difficult to choose the universe of discourse and the range of each membership function used. For adjusting the range of membership functions, series gains are used with the inputs and the output of the fuzzy controller. These gains are optimized using Genetic algorithm (GA) and particle swarm optimization (PSO) as Figure 5 illustrates.

These gains are optimized based on the genetic algorithm and the particle swarm optimization techniques to maximize the output DC power *P* along the simulation period $t_{s}$, which maximize the tracking efficiency. The objective function $F_{obj}$ is presented in (8) and given the maximum output DC power, $P_{MP}$, according to the specifications of the PV panel/array, the tracking efficiency is presented by (9) [29].

(8) $$F_{obj}=\int_{0}^{t_{s}}P\;dt$$

(9) $$\eta_{tracking}=\frac{\int_{0}^{t_{s}}P\;dt}{\int_{0}^{t_{s}}P_{MP}\;dt}$$

**Figure 6:**
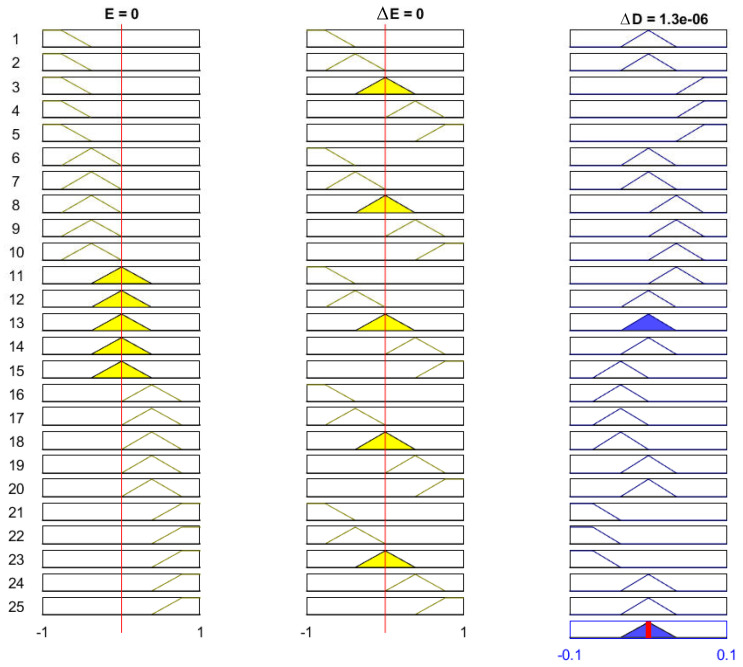
The 25 fuzzy rules (If-Then rules), which relates the inputs and the outputs of FLC system.

The rule base is presented in Table 1, from which the output of the fuzzy logic system ΔD is generated based on the inputs *E* and ΔE. The notations in this table are; PB: positive big, PS: positive small, ZE: zero, NS: negative small, NB: negative big. Figure 6 shows the 25 rules (If-Then rules), which relates the inputs and the output of the FLC system.

#### 2.3.2. GA-ANN for MPPT

The artificial neural networks (ANNs) have the advantage of accurately replacing complex mathematical models [48]. The ANNs are exploited in the MPPT systems as it can offer accurate and fast tracking as the PV system is subjected to various environmental conditions [35].

**Figure 7:**
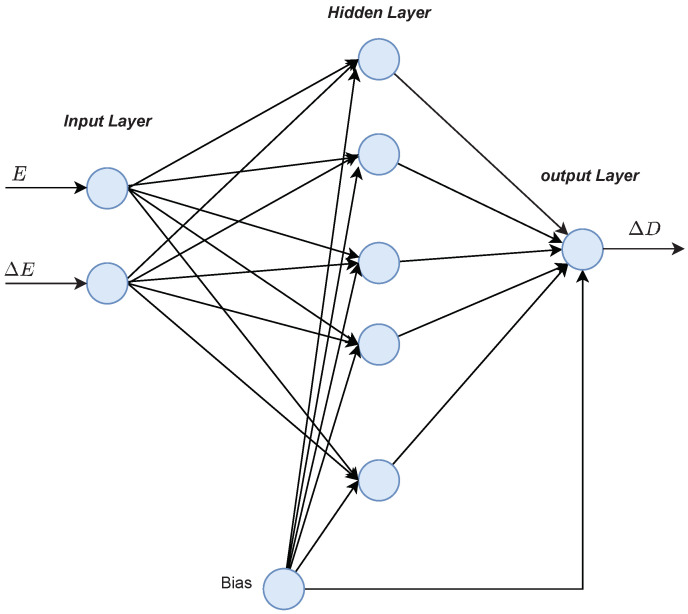
The proposed design of the artificial neural network (ANN) used for maximum power point tracking (MPPT).

A proposed design of an artificial neural network used for MPPT is presented in Figure 7, where the proposed inputs *E* and ΔE have a privileged response over that of the traditional inputs *G* and $T_{c}$ [40]. The ANN output is the change of duty cycle (ΔD), which is utilized to adjust the PV voltage through the DC-DC boost converter.

The manual choice of the best ANN architecture to provide the best response for MPPT is tedious. The important parameters of ANN design are the number of hidden layers, the number of neurons in each hidden layer, the type of the activation function and the type of the learning algorithm. To acquire the optimized design of the ANN, GA optimization is used for simultaneous adjusting of these parameters based on a selected objective function. The training patterns used for learning the ANN are obtained at different operating *G* and $T_{c}$ from the theoretical model presented in Section 2.1. The objective function used for this optimization process is the mean square error (MSE) between the target and output of the ANN for all the training patterns. The objective function used is given as [40]:

(10) $$Obj_{F}\left(v_{n}\right)=\frac{1}{N}\sum_{p=1}^{N}{\left(t_{p}-o_{p}\left(v_{n}\right)\right)}^{2}$$

where, $Obj_{F}$ is the objective function, *N* is the number of training patterns, $t_{p}$ is the target of pattern *p*, $o_{p}$ is the actual output of pattern *p* and $v_{n}$ is the optimized set of parameters. The ANN parameters optimized are the number neurons in the hidden layer, the learning algorithm and the type activation function. For the number neurons in the hidden layer, the optimization process select a number from 1 to 30 neurons in one hidden layer. For the learning algorithm, it is chosen from three types, which are the Levenberg-Marquardt algorithm (trainlm), the gradient descent with momentum (traingdm) and the scaled conjugate gradient (trainscg). For the activation function, it is selected from three types for the neurons in each layer, which are the linear (purlin), the hyperbolic tangent sigmoid (tansig) and the logistic sigmoid (logsig).

## 3. Application of the Artificial Intelligence Methods for MPPT

**Figure 8:**
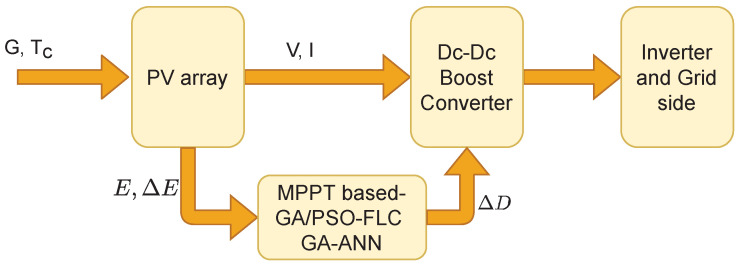
A schematic diagram of the grid-connected PV system.

To illustrate the improvement achieved when the AI based methods are used for MPPT, a grid connected PV model is used for the application of these methods. The model used is a modification of the 100-kW grid-connected PV array model in MATLAB. This PV array is composed of 5 parallel strings with each string consists of 66 series panel of type SUNPOWER 305. A schematic diagram of the PV model is shown in Figure 8. The system comprises a PV array, a boost converter, an inverter and the grid. A control system, which represents the MPPT system is used to adjust the duty cycle, which consequently adjusts the PV voltage to reach the optimal operating point. This control system contains the AI based MPPT method, e.g., GA-FLC, PSO-FLC, GA-ANN. A voltage source converter (VSC) control is used for the optimal inverter operation, therefore the variation of the inverter voltage is insignificant in this study.

**Figure 9:**
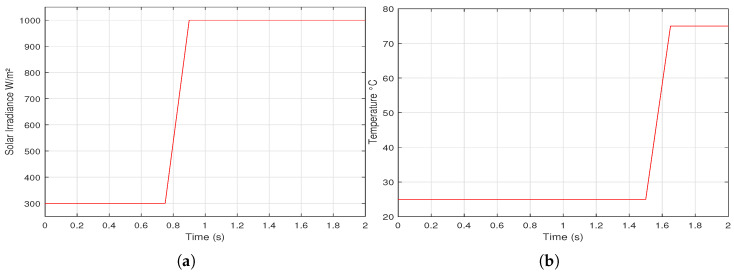
A proposed linear variation of: (**a**) solar irradiance (**b**) cell temperature.

**Figure 10:**
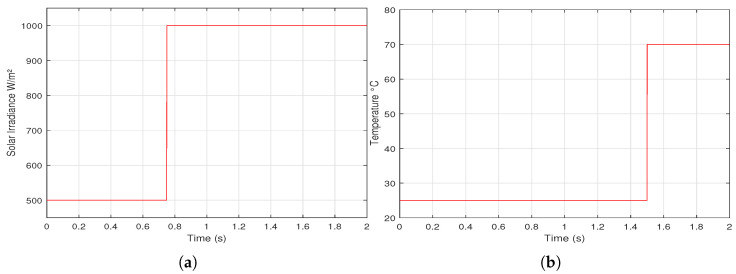
A proposed step variation of: (**a**) solar irradiance (**b**) cell temperature.

To check different MPPT methods, two scenarios of environmental variations of *G* and $T_{c}$, linear and step variation, are proposed as shown in Figure 9 and Figure 10, respectively.

### 3.1. Application of GA/PSO-FLC Based MPPT Method

**Figure 11:**
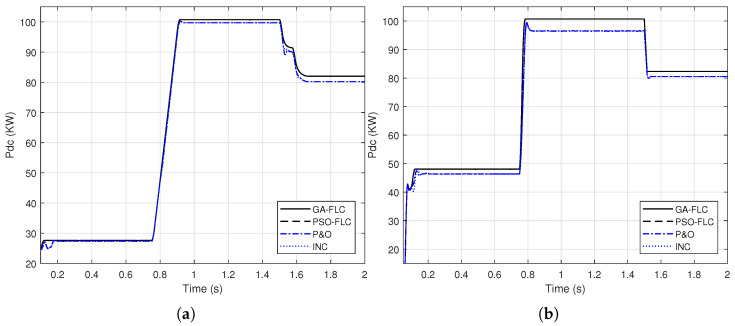
A comparison of the output DC power of the PV array using genetic algorithm (GA)-FLC, particle swarm optimization (PSO)-FLC, perturb-and-observe (P&O) and incremental conductance (INC) for; (**a**) linear variation of *G* and $T_{c}$ (**b**) step variation of *G* and $T_{c}$.

In this section, the FLC system, presented in Section 2.3.1, is optimized using the genetic algorithm and the particle swarm optimization to adjust the three gains, $K_{E}$, $K_{\Delta E}$ and $K_{\Delta D}$. Based on the objective function presented in (8), the GA optimized gains are found to be; $K_{E}=0.001817$, $K_{\Delta E}=0.0086864$, $K_{\Delta D}=19.2681$, where these resulted from PSO optimization are; $K_{E}=0.001695$, $K_{\Delta E}=0.0089926$, $K_{\Delta D}=21.4414$. To testify the effectiveness of these parameters to provide improved response of the output DC power, the responses are presented in Figure 11 compared to the responses of the conventional methods presented in Section 2.2.

It can be shown from Figure 11, that the response of the GA-FLC and PSO-FLC MPPT methods are comparable and these of the P&O and INC are also comparable. However, the improvement of the FLC based MPPT over that of the conventional methods is obvious.

**Figure 12:**
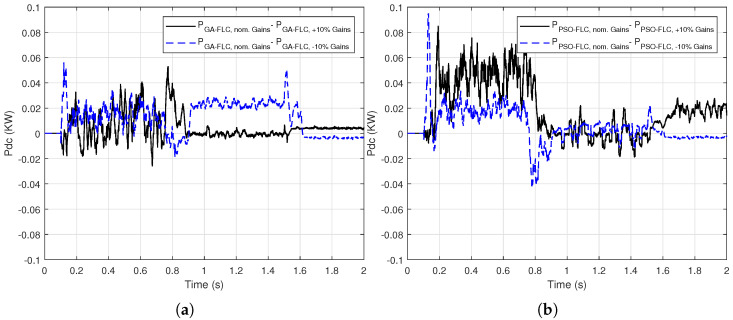
The output DC power difference when changing the optimized gains with ±10% of: (**a**) GA-FLC based MPPT method (**b**) PSO-FLC based MPPT method for linear variation of *G* and $T_{c}$.

To show that the optimized gains are “non-fragile”, these gains are subjected to ±10% change to examine their effect on the output DC power response of the proposed GA/PSO-FLC MPPT method for linear variation of *G* and $T_{c}$. Figure 12 presents the error in the output DC power for GA-FLC and PSO-FLC based methods, which illustrates the power difference when the gains change by ±10% referred to the nominal gains case. It is shown that this error ranges from −0.02 to 0.06 KW for GA-FLC method and from −0.04 to 0.1 KW for PSO-FLC method. These error values emphasize the non-fragility of the optimized gains of the FLC based MPPT method. Note that the swarm size is 100 and the number of iterations is 50 for the PSO. Regarding GA, the number of populations is 100 and the number of iterations is 50. We use the parameters of the published GA and PSO optimizers.

### 3.2. Application of GA-ANN MPPT Method

**Figure 13:**
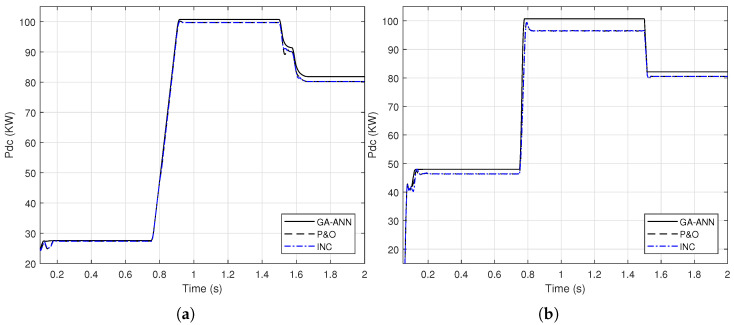
A comparison of the output DC power of the PV array using GA-ANN, P&O and INC for; (**a**) linear variation of *G* and $T_{c}$ (**b**) step variation of *G* and $T_{c}$.

This section introduces the application of GA-ANN based MPPT system to the PV model. As presented in Section 2.3.2, the ANN architecture parameters are optimized using the genetic algorithm. Based on the objective function presented in (10), the optimized number of neurons in the hidden layer is 28 neurons, whereas the optimized activation functions are the purlin for the neurons in the hidden layer and the logsig for the neuron in the output layer, while the optimized learning algorithm is the traingdm [40]. To show the effectiveness of the GA-ANN based MPPT, its response of the output DC power compared to these of the conventional methods, is presented in Figure 13.

It is shown from Figure 13, that the improvement in the response of the GA-ANN based MPPT method compared to these of the P&O and INC is apparent. Putting Figure 11 and Figure 13 in perspective, arises the question of which response is better, the ANN based or the FLC based MPPT method for this PV system.

A sample for validating these simulation results is given as follows. The output DC power of the SUNPOWER panel provided by the manufacture is 305 W at standard test conditions. The PV array consists of 66 parallel strings, each string has 5 series panels, which results in a total power of 100.7 KW at G=1000 W/m${}^{2}$ and $T_{c}=25^{\;\circ }$C. Comparing this power with the output DC power obtained from the simulation results in Figure 11 and Figure 13 implies the agreement of the simulated model with the manufacture data.

### 3.3. Comparison of GA/PSO-FLC and GA-ANN Based MPPT

**Figure 14:**
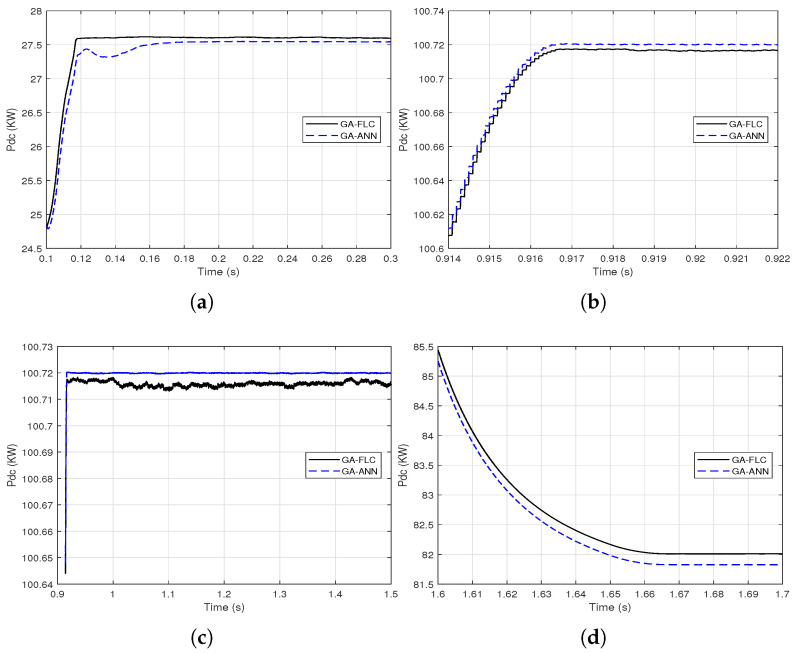
Proximate views of the output DC power of the PV array when using the GA-FLC and the GA-ANN based MPPT methods for linear variations of *G* and $T_{c}$: (**a**) from 0.1 to 0.3 s; (**b**) from 0.914 to 0.922 s; (**c**) from 0.9 to 1.5 s; (**d**) from 1.6 to 1.7 s.

**Figure 15:**
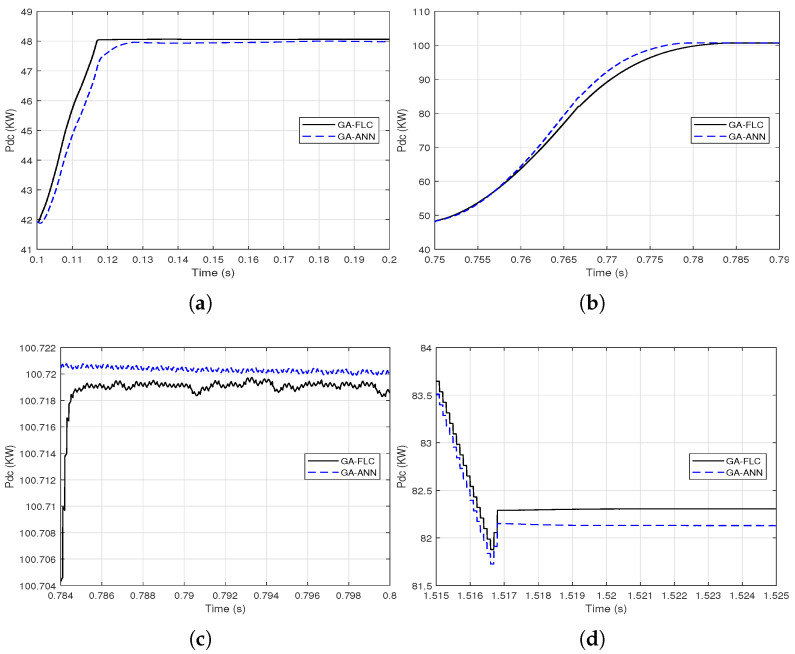
Proximate views of the output DC power of the PV array when using the GA-FLC and the GA-ANN based MPPT methods for step variations of *G* and $T_{c}$: (**a**) from 0.1 to 0.2 s; (**b**) from 0.75 to 0.79 s; (**c**) from 0.784 to 0.8 s; (**d**) from 1.515 to 1.525 s.

As the responses of the output DC power of the PV array when using GA-FLC and GA-ANN based MPPT are apparently comparable, proximate comparisons are presented at different time periods as shown in Figure 14 and Figure 15.

**Figure 16:**
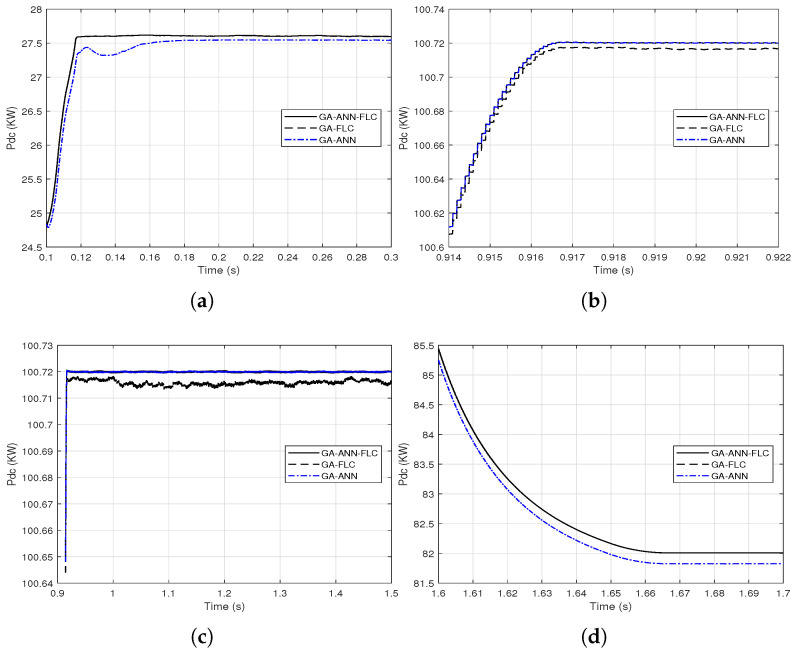
Close views of combining the output DC power response from the GA-FLC and the GA-ANN based MPPT methods based on the environmental conditions *G* and $T_{c}$ for their linear variations: (**a**) from 0.1 to 0.3 s; (**b**) from 0.914 to 0.922 s; (**c**) from 0.9 to 1.5 s; (**d**) from 1.6 to 1.7 s.

**Figure 17:**
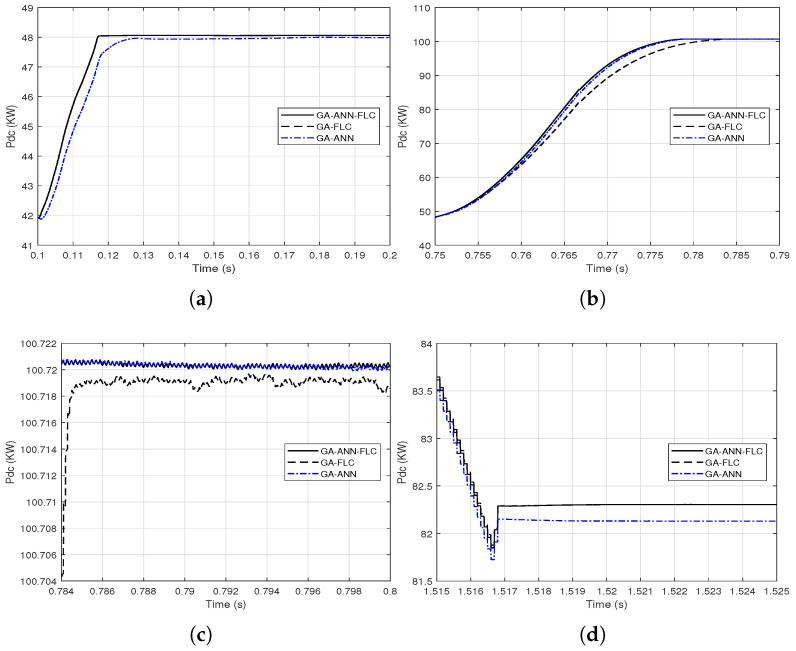
Close views of combining the output DC power response from the GA-FLC and the GA-ANN based MPPT methods based on the environmental conditions *G* and $T_{c}$ for their step variations (**a**) from 0.1 to 0.2 s; (**b**) from 0.75 to 0.79 s; (**c**) from 0.784 to 0.8 s; (**d**) from 1.515 to 1.525 s.

It is clear from Figure 14 and Figure 15 that the GA-FLC based MPPT method has a privileged response over that of GA-ANN based method in case of low *G* and high $T_{c}$. However, the GA-ANN based method has its privilege in case of the high *G* and low $T_{c}$. Therefore, a wise switching between the two responses is proposed, according to the values of *G* and $T_{c}$, to exploit the best of the two methods. Explicitly, at high *G* and low $T_{c}$, the used MPPT method is the GA-ANN, otherwise the GA-FLC is used. Figure 16 and Figure 17 presented an illustration of the behavior of three MPPTs methods, which are the GA-ANN, the GA-FLC and the merged MPPT method, which is referred as GA-FLC-ANN. It is illustrated that the reponse of the GA-FLC-ANN almost coincides with the higher DC power at all values of *G* and $T_{c}$.

A quantitative comparison of the conventional and AI based MPPT methods is given in Table 2 in terms of the PV array output energy over the simulation period and the tracking speed expressed by the rise time.

As this table summarizes, the improvements of the AI based MPPT methods over the conventional methods, in terms of output energy and tracking speed, are apparent. The GA-FLC and PSO-FLC based MPPT are almost the same. An improvement of the GA-FLC based MPPT over that of GA-ANN regarding the output energy, while the GA-ANN is superior regarding the tracking speed. The combination of the GA-FLC and GA-ANN exploits the best of each of them from both the output energy and tracking speed.

### 3.4. Dynamic Environmental Conditions Test of the AI Based Methods

**Figure 18:**
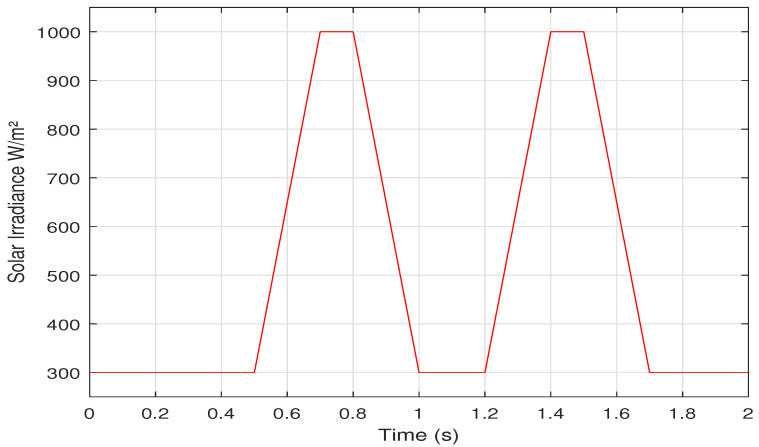
A dynamic solar irradiance change based on EN50530 standard.

In this section the proposed AI based methods are tested under dynamic environmental conditions based on EN50530 standard [49,50]. One sequence of the dynamic test EN50530 is presented, in which the solar irradiance changes from 300 W/m${}^{2}$ to 1000W/m${}^{2}$, with $T_{c}=25^{\;\circ }$C, as shown in Figure 18.

**Figure 19:**
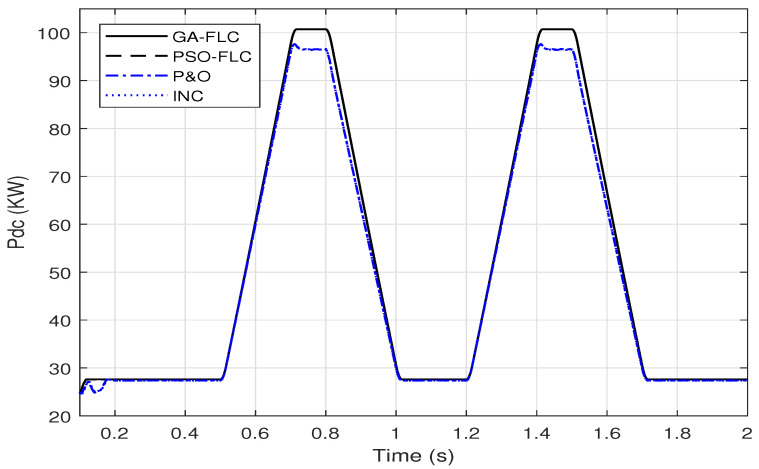
A Comparison of the output DC power of the PV array using GA-FLC, PSO-FLC, P&O and INC MPPT methods for a dynamic irradiance change based on EN50530 standard.

To evaluate the proposed GA/PSO-FLC tracking system under these environmental dynamic change, Figure 19 presents a comparison of the response of the output dc power with these of the conventional MPPT methods.

Figure 19 illustrates that the GA-FLC based MPPT method behaves almost like the PSO-FLC MPPT method as presented in Section 3.1. The improvement in output DC power responses of the GA-FLC and PSO-FLC MPPT over these of the P&O and INC are apparent.

**Figure 20:**
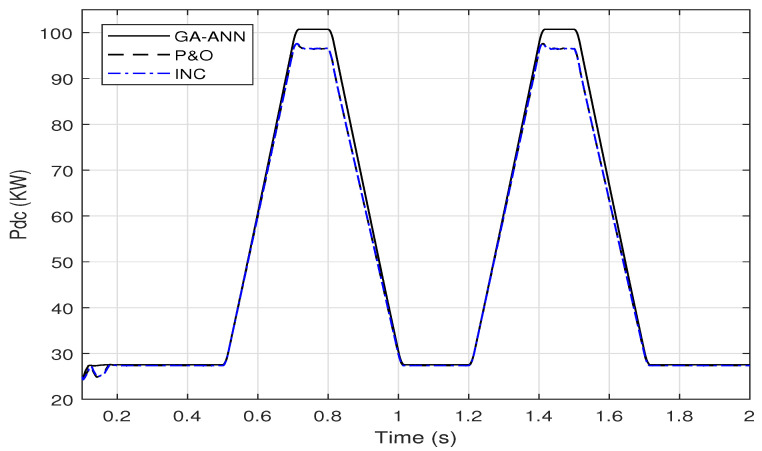
A Comparison of the output DC power of the PV array using GA-ANN, P&O and INC MPPT methods for a dynamic irradiance change based on EN50530 standard.

Figure 20 shows also the superiority of the output DC power response of the GA-ANN based MPPT system over these of the conventional methods.

**Figure 21:**
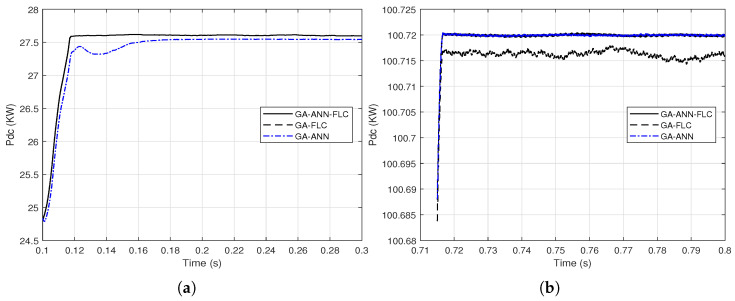
Proximate views of combining the responses of the output DC power from the GA-FLC and the GA-ANN based MPPT methods for the dynamic solar irradiance change (**a**) from 0.1 to 0.3 s; (**b**) from 0.71 to 0.8 s.

As remarked in Section 3.3 that the GA-FLC based MPPT method has a superior DC output power response over that of GA-ANN method in case of low *G* and for high *G*, the GA-ANN tracking method is better. Therefore, a combination between the two responses provides the maximum harvest of the output DC power from the PV array. Figure 21 presents proximate views of the output DC power response when combining the GA-FLC and GA-ANN methods at different time periods.

### 3.5. Discussion

The presented AI based MPPT methods show a considerable improvement of the output DC power in terms of its magnitude and the fast tracking when the environmental conditions change. As the FLC has a lot of success in many fields, it is proposed for the purpose of MPPT systems. The key of success of the FLC based MPPT is the well adjustment of the membership functions used. In this paper, a genetic algorithm and a particle swarm optimization techniques are used to adjust the width of membership functions through optimizing the series gains of the inputs and output. The two optimization techniques provide comparable values of gains and therefore the response of the MPPT system is comparable. The GA-FLC and the PSO-FLC provide superior response of output DC power over the conventional methods, e.g., the incremental conductance and the perturb and observe methods. However, the genetic algorithm based FLC has a superior response as MPPT system over that of the particle swarm optimization. The artificial neural network method has its effective footprint as a MPPT system. However, the choice of its architecture, i.e., the number of neurons in the hidden layers, the learning algorithm and the activation function of each neuron, is tedious. This paper proposes an optimization technique to choose from certain variety of options to provide an optimum design of the architecture of the ANN to be suitable for MPPT. The genetic algorithm is successfully used for this purpose. The comparison of the GA-FLC and GA-ANN based MPPT systems reveals that the latter one has its response advantage over the former for high solar irradiance and low temperature. Conversely, the GA-FLC based MPPT has its superior response over that of the GA-ANN for low solar irradiance and high temperature. Therefore, a combination of the GA-FLC and GA-ANN is proposed which exploits each of them where its response is superior. Although the combination of the GA-FLC and GA-ANN provides a small improvement in both magnitude of output energy and tracking speed, this improvement is for a time period of 2 s, which gives only a sample of the promising improvement for longer periods. Although the improvement achieved with these AI based MPPT methods, there still more complicated scenarios to be to tested, which will be covered as a future work. These complicated conditions include partial shading of some PV panels and testing the response of the PV array during dynamic conditions, such as short circuits or load changes.

The MPPT system is used in our model for adjusting the PV array voltage via controlling the duty cycle, which is used by the pulse width modulation for the dc-dc boost converter. The inverter is controlled in the model via the VSC control system to adjust the inverter operation and keep a nearly constant dc-link voltage. An example for industrial PV inverter is Fronius Symo 15.0-3-M which has a max efficiency (PV grid) of 98.1% [51]. The dc-dc boost converter can adjust the PV array voltage based on the constant dc-link voltage. Note that we do not aim to improve the inverter efficiency but enhance the MPPT based solar charge controller. In Table 2, we present a comparison of the output energy of each method (for a small scale of time), which is an indication of the economic benefits. The more harvesting PV energy, the more the economical benefits from the PV system. In recent years all the MPPT system, used by large manufacturers (like Fronius International GmbH or SMA Solar Technology AG), are microprocessor-based controllers. The proposed AI-based algorithm can be programmed to any microcontroller and applied to a practical system in general.

## 4. Conclusions

The increase of the tracking efficiency is essential to increase the overall efficiency of the PV system. The conventional MPPT methods are used to keep the optimal values of the output DC power and voltage. The artificial intelligence methods can, efficiently, replace or support these conventional methods. The fuzzy logic controller are used successfully for MPPT with the aid of genetic algorithm/particle swarm optimization techniques to adapt the range of membership functions through series gains. The artificial neural networks are also used efficiently as MPPT system with enhancing its architecture using genetic algorithm. The simulation results demonstrate that the GA/PSO-FLC and the GA-ANN based MPPT methods have significant improvement in term of the output DC power and the tracking speed. The GA-FLC and GA-ANN based MPPT methods are merged according to the environmental conditions to propose a general AI based MPPT method with ameliorated performance. Although, these AI based methods are introduced for this grid connected PV model, the strategy of these methods can be applied in different applications for stand-alone PV systems. These methods can help for harvesting the maximum possible output power from PV arrays for different applications such as charging electric vehicles and irrigation purposes. More investigations will be presented as future work for emphasizing the effectiveness of the proposed strategy for different applications.

## Figures and Tables

**Table 1:** Rule base of FLC with the inputs *E* and ΔE and the output ΔD.

ΔE *E*	NB	NS	ZE	PS	PB
**NB**	ZE	ZE	PB	PB	PB
**NS**	ZE	ZE	PS	PS	PS
**ZE**	PS	ZE	ZE	ZE	NS
**PS**	NS	NS	NS	ZE	ZE
**PB**	NB	NB	NB	ZE	ZE

**Table 2:** A quantitative evaluation of the proposed MPPT methods in terms of the produced energy and the rise time.

	Step Variations of G and $T_{c}$		Ramp Variations of G and $T_{c}$
	Output Energy (KJ)	Rise Time (s)	Output Energy (KJ)
INC	141.92	0.0251	127.52
P&O	141.95	0.0239	127.54
GA-FLC	147.27	0.0193	129.43
PSO-FLC	147.26	0.0193	129.43
GA-ANN	147.17	0.0169	129.31
COMBINED GA-FLC-ANN	147.34	0.0168	129.44

## Data Availability

The data presented in this study are available on request from the corresponding author.

## References

[1] Böök H., Lindfors A.V. (2020). Site-specific adjustment of a NWP-based photovoltaic production forecast. Sol. Energy.

[2] Cross S., Hast A., Kuhi-Thalfeldt R., Syri S., Streimikiene D., Denina A. (2015). Progress in renewable electricity in Northern Europe towards EU 2020 targets. Renew. Sustain. Energy Rev..

[3] Khosravi A., Olkkonen V., Farsaei A., Syri S. (2020). Replacing hard coal with wind and nuclear power in Finland-impacts on electricity and district heating markets. Energy.

[4] Eltamaly A.M., Abdelaziz A.Y. (2019). Modern Maximum Power Point Tracking Techniques for Photovoltaic Energy Systems.

[5] Mahmoud K., Lehtonen M. (2020). Three-level control strategy for minimizing voltage deviation and flicker in PV-rich distribution systems. Int. J. Electr. Power Energy Syst..

[6] Mansour D.E.A., Abdel-Gawad N.M.K., El Dein A.Z., Ahmed H.M., Darwish M.M.F., Lehtonen M. (2020). Recent Advances in Polymer Nanocomposites Based on Polyethylene and Polyvinylchloride for Power Cables. Materials.

[7] Chenouard R., El-Sehiemy R.A. (2020). An interval branch and bound global optimization algorithm for parameter estimation of three photovoltaic models. Energy Convers. Manag..

[8] Abbas A.S., El-Sehiemy R.A., Abou El-Ela A., Ali E.S., Mahmoud K., Lehtonen M., Darwish M.M. (2021). Optimal Harmonic Mitigation in Distribution Systems with Inverter Based Distributed Generation. Appl. Sci..

[9] Bayoumi A.S., El-Sehiemy R.A., Mahmoud K., Lehtonen M., Darwish M.M.F. (2021). Assessment of an Improved Three-Diode against Modified Two-Diode Patterns of MCS Solar Cells Associated with Soft Parameter Estimation Paradigms. Appl. Sci..

[10] Pazikadin A.R., Rifai D., Ali K., Mamat N.H., Khamsah N. (2020). Design and Implementation of Fuzzy Compensation Scheme for Temperature and Solar Irradiance Wireless Sensor Network (WSN) on Solar Photovoltaic (PV) System. Sensors.

[11] Abouelatta M.A., Ward S.A., Sayed A.M., Mahmoud K., Lehtonen M., Darwish M.M.F. (2020). Fast Corona Discharge Assessment Using FDM integrated With Full Multigrid Method in HVDC Transmission Lines Considering Wind Impact. IEEE Access.

[12] Mahmoud K., Lehtonen M. (2019). Simultaneous allocation of multi-type distributed generations and capacitors using generic analytical expressions. IEEE Access.

[13] Ali M.N. (2015). Fuzzy Logic PSS Assisted by Neighboring Signals to Mitigate the Electromechanical Wave Propagation in Power Systems. Telkomnika Indones. J. Electr. Eng..

[14] Ali M.N. (2021). A Novel Combination Algorithm of Different Methods of Maximum Power Point Tracking for Grid-Connected Photovoltaic Systems. J. Sol. Energy Eng..

[15] Sera D., Mathe L., Kerekes T., Spataru S.V., Teodorescu R. (2013). On the Perturb-and-Observe and Incremental Conductance MPPT Methods for PV Systems. IEEE J. Photovoltaics.

[16] Subudhi B., Pradhan R. (2013). A comparative study on maximum power point tracking techniques for photovoltaic power systems. IEEE Trans. Sustain. Energy.

[17] Seyedmahmoudian M., Horan B., Soon T.K., Rahmani R., Oo A.M.T., Stojcevski S.M.A. (2016). State of the art artificial intelligence-based MPPT techniques for mitigating partial shading effects on PV systems A review. Renew. Sustain. Energy Rev..

[18] Ramaprabha R., Gothandaraman V., Kanimozhi K., Divya R., Mathur B.L. Maximum power point tracking using GA-optimized artificial neural network for Solar PV system. Proceedings of the 1st International Conference on Electrical Energy Systems (ICEES).

[19] Rezk H., Aly M., Al-Dhaifallah M., Shoyama M. (2019). Design and Hardware Implementation of New Adaptive Fuzzy Logic-Based MPPT Control Method for Photovoltaic Applications. IEEE Access.

[20] Alajmi B.N., Ahmed K.H., Finney S.J., Williams B.W. (2011). Fuzzy-logic-control approach of a modified hill-climbing method for maximum power point in microgrid standalone photovoltaic system. IEEE Trans. Power Electron..

[21] Titri S., Larbes C., Toumi K.Y., Benatchba K. (2017). A new MPPT controller based on the Ant colony optimization algorithm for Photovoltaic systems under partial shading conditions. Appl. Soft Comput..

[22] Liu Y.H., Huang S.C., Huang J.W., Liang W.C. (2012). A Particle Swarm Optimization-Based Maximum Power Point Tracking Algorithm for PV Systems Operating Under Partially Shaded Conditions. IEEE Trans. Energy Convers..

[23] Joisher M., Singh D., Taheri S., Espinoza-Trejo D.R., Pouresmaeil E., Taheri H. (2020). A Hybrid Evolutionary-Based MPPT for Photovoltaic Systems Under Partial Shading Conditions. IEEE Access.

[24] Nugraha D.A., Lian K.L. (2019). A Novel MPPT Method Based on Cuckoo Search Algorithm and Golden Section Search Algorithm for Partially Shaded PV System. Can. J. Electr. Comput. Eng..

[25] Padmanaban S., Priyadarshi N., Bhaskar M.S., Holm-Nielsen J.B., Hossain E., Azam F. (2019). A Hybrid Photovoltaic-Fuel Cell for Grid Integration With Jaya-Based Maximum Power Point Tracking: Experimental Performance Evaluation. IEEE Access.

[26] Huang C., Wang L., Zhang Z., Shun-cheung Yeung R., Bensoussan A., Shu-hung Chung H. (2020). A Novel Spline Model Guided Maximum Power Point Tracking Method for Photovoltaic Systems. IEEE Trans. Sustain. Energy.

[27] Çelik Ö., Teke A. (2017). A Hybrid MPPT method for grid connected photovoltaic systems under rapidly changing atmospheric conditions. Electr. Power Syst. Res..

[28] Zamora A.C., Vazquez G., Sosa J., Martinez-Rodriguez P.R., Juarez M.A. Efficiency based comparative analysis of selected classical MPPT methods. Proceedings of the IEEE International Autumn Meeting on Power, Electronics and Computing (ROPEC).

[29] Bendib B., Belmili H., Krim F. (2015). A survey of the most used MPPT methods: Conventional and advanced algorithms applied for photovoltaic systems. Renew. Sustain. Energy Rev..

[30] Rezk H., Eltamaly A.M. (2015). A comprehensive comparison of different MPPT techniques for photovoltaic systems. Sol. Energy.

[31] Elsisi M., Mahmoud K., Lehtonen M., Darwish M.M.F. (2021). An Improved Neural Network Algorithm to Efficiently Track Various Trajectories of Robot Manipulator Arms. IEEE Access.

[32] Elsisi M., Mahmoud K., Lehtonen M., Darwish M.M.F. (2021). Reliable Industry 4.0 Based on Machine Learning and IoT for Analyzing, Monitoring, and Securing Smart Meters. Sensors.

[33] Elsisi M., Tran M.Q., Mahmoud K., Lehtonen M., Darwish M.M.F. (2021). Deep Learning-Based Industry 4.0 and Internet of Things Towards Effective Energy Management for Smart Buildings. Sensors.

[34] Liu Y.H., Liu C.L., Huang J.W., Chen J.H. (2013). Neural-network-based maximum power point tracking methods for photovoltaic systems operating under fast changing environments. Sol. Energy.

[35] Bahgat A., Helwa N., Ahmad G., El Shenawy E. (2005). Maximum power point traking controller for PV systems using neural networks. Renew. Energy.

[36] Rizzo S.A., Scelba G. (2015). ANN based MPPT method for rapidly variable shading conditions. Appl. Energy.

[37] Elobaid L.M., Abdelsalam A.K., Zakzouk E.E. (2015). Artificial neural network-based photovoltaic maximum power point tracking techniques: A survey. IET Renew. Power Gener..

[38] Kulaksız A.A., Akkaya R. (2012). A genetic algorithm optimized ANN-based MPPT algorithm for a stand-alone PV system with induction motor drive. Sol. Energy.

[39] Abu Eldahab Y.E., Saad N.H., Zekry A. (2014). Enhancing the maximum power point tracking techniques for photovoltaic systems. Renew. Sustain. Energy Rev..

[40] Nour Ali M. Improved Design of Artificial Neural Network for MPPT of Grid-Connected PV Systems. Proceedings of the 2018 Twentieth International Middle East Power Systems Conference (MEPCON).

[41] Ahmed E., Mahmoud A., Fahmy B., Wagdy M. (2019). Adaptive Under Frequency Load Shedding Scheme Using Genetic Algorithm Based Artificial Neural Network. J. Electr. Electron. Eng..

[42] Villalva M.G., Gazoli J.R., Filho E.R. (2009). Comprehensive approach to modeling and simulation of photovoltaic arrays. IEEE Trans. Power Electron..

[43] Femia N., Petrone G., Spagnuolo G., Vitelli M. (2012). Power Electronics and Control Techniques for Maximum Energy Harvesting in Photovoltaic Systems.

[44] Esram T., Chapman P.L. (2007). Comparison of photovoltaic array maximum power point tracking techniques. IEEE Trans. Energy Convers..

[45] Sivanandam S., Sumathi S., Deepa S. (2007). Introduction to Fuzzy Logic Using MATLAB.

[46] Bounechba H., Bouzid A., Nabti K., Benalla H. (2014). Comparison of perturb & observe and fuzzy logic in maximum power point tracker for PV systems. Energy Procedia.

[47] Chen Y.T., Jhang Y.C., Liang R.H. (2016). A fuzzy-logic based auto-scaling variable step-size MPPT method for PV systems. Sol. Energy.

[48] Haykin S. (1999). Neural Networks: A Comprehensive Foundation.

[49] Li X., Wen H. Evaluation of different Maximum power point tracking techniques by using EN 50530 dynamic test standard. Proceedings of the 2016 IEEE International Conference on Power Electronics, Drives and Energy Systems (PEDES).

[50] Andrejašič T., Jankovec M., Topič M. (2011). Comparison of direct maximum power point tracking algorithms using EN 50530 dynamic test procedure. IET Renew. Power Gener..

[51] FRONIUS SYMO. https://www.fronius.com/en-gb/uk/photovoltaics/products/all-products/inverters/fronius-symo/fronius-symo-15-0-3-m.

